# From Eye Drops to ICU, a Case Report of Three Side Effects of Ophthalmic Timolol Maleate in the Same Patient

**DOI:** 10.1155/2015/714919

**Published:** 2015-08-06

**Authors:** Muhammad Asim Rana, Ahmed Fouad Mady, Basheer Abdel Rehman, Abdulrahman Alharthy, Basim Huwait, Asim Riaz, Waleed Tharwat Aletreby

**Affiliations:** ^1^King's Mill Hospital, Nottinghamshire NG17 4JL, UK; ^2^Department of Intensive Care Medicine, King Saud Medical City, Riyadh 11373, Saudi Arabia

## Abstract

Timolol Maleate (also called Timolol) is a nonselective beta-adrenergic blocker and a class II antiarrhythmic drug, which is used to treat intraocular hypertension. It has been reported to cause systemic side effects especially in elderly patients with other comorbidities. These side effects are due to systemic absorption of the drug and it is known that Timolol is measurable in the serum following ophthalmic use. Chances of life threatening side effects increase if these are coprescribed with other cardiodepressant drugs like calcium channel or systemic beta blockers. We report a case where an elderly patient was admitted with three side effects of Timolol and his condition required ICU admission with mechanical ventilation and temporary transvenous pacing. The case emphasizes the need of raising awareness among physicians of such medications about the potential side effects and drug interactions. A close liaison among patient's physicians is suggested.

## 1. Case Report

**Figure 1:**
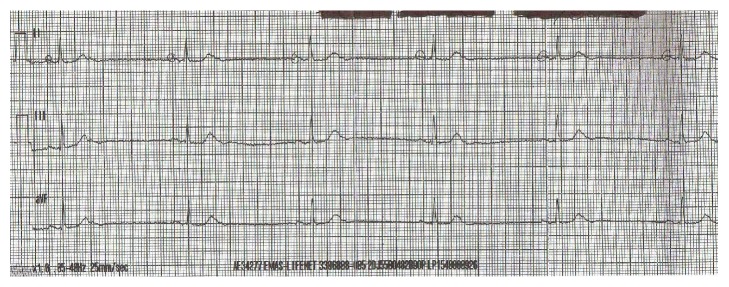
ECG showing sinus bradycardia.

**Figure 2:**
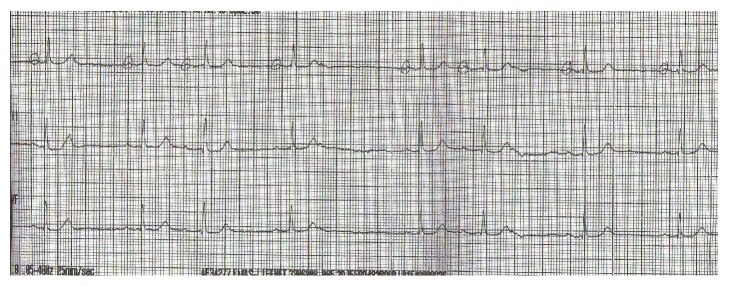
ECG with sinus dysfunction and Mobitz 1 block.

An 84-year-old male patient with past medical history consisting of hypertension, hypercholesterolemia, type 2 diabetes mellitus, and open angle glaucoma was admitted to ICU via A & E with an unwitnessed collapse and decreased level of consciousness. In emergency department (ED) his GCS was recorded as 5/15, blood sugar was found to be only 34 mg/dL, his heart rate was 34 beats per minutes (bpm), and BP was 58/43 mm Hg. His ECG showed sinus bradycardia with variable blocks including sinus node dysfunction and type 1 Mobitz heart block pattern (Figures 1 and 2). He was intubated and ventilated in ED and given dextrose 50% 100 mL and 0.5 mg Atropine. His heart rate rose to 88 bpm and with improvement of heart rate and correction of blood sugar his BP became 135/88 and his GCS improved to 13/15. Unfortunately, heart rate started to drop again to 40 s and blood sugar showed a downward trend for which he was given 1 mg glucagon and was transferred to ICU. By the time he reached ICU his heart rate had improved to 90 bpm. In ICU after 30 minutes his heart rate dropped again so he was given another dose of Atropine and a temporary transvenous pace maker (TPM) was inserted. The patient's blood sugar also improved temporarily but eventually required intravenous infusion of 10% dextrose for the next 12 hours before it was stabilized. His GCS improved to 14/15 and he was extubated the next day 16 hours after admission but repeated attempts to switch off his pace maker revealed underlying brady-arrythmias with heart rate dropping to 34–40 bpm associated with presyncopal symptoms comprising of deterioration in attention and episodes of drowsiness. The pace maker was eventually switched off after 26 hours of observation when no more episodes of bradycardia were observed.

A detailed neurological assessment was carried out after extubation which showed the patient to be confused with no motor deficit. His CT brain was done which showed no abnormality and a detailed history was sought from the patient's wife who pointed out that the patient has been behaving in a weird and confused way for the last 5 days but his wife attributed that to demise of one of his close relative who died a week ago. She further added that he stopped taking his oral medications 2 days before he was admitted but he was given his eye drops regularly by his daughter who feared blindness in case the medication was not given. His drug history consisted of Enalapril 10 mg OD, Atorvastatin 10 mg OD, Metformin 500 mg BID, and Timolol Maleate 0.5% one drop each eye twice a day which was started 35 days before admission to ICU when he was diagnosed as a case of glaucoma.

Tilt table test was not carried out and keeping in view his improvement in blood sugar and heart rate after administration of glucagon his clinical picture of hypoglycemia, confusion, and bradyarrhythmia was recognized to be because of ophthalmic Timolol Maleate which was changed to Travoprost (a prostaglandin F2 analogue) after consulting the ophthalmologist.

The patient was kept under observation in ICU for another 24 hours. His intrinsic heart rate improved to 82 bpm and his TPM wire was removed before transferring to ward. Patient's confusion also improved gradually over the next 3 days and he was discharged on Enalapril, Travoprost, and Metformin. His followup for the next two months was unremarkable.

## 2. Discussion

Timolol was first introduced in 1978. It is a nonselective beta-adrenergic antagonist. It blocks both beta1 and beta2 adrenergic receptors. In the eye drop form, it has been the main stay of treatment in glaucoma and raised intraocular pressure secondary to traumatic cataract in 80 s and 90 s [1–3].

In general, beta-adrenergic blocking agents reduce cardiac output both in healthy subjects and patients with heart diseases. In patients with severe impairment of myocardial function, beta-adrenergic receptor blocking agents may inhibit sympathetic stimulatory effect necessary to maintain adequate cardiac function. Pharmacokinetics and pharmacodynamics of Timolol Maleate have been studied extensively. When given orally, Timolol is well absorbed and undergoes considerable first pass metabolism by cytochrome P450 2D6 enzyme system (CYP 2D6). Plasma half-life of Timolol is approximately 4 hours and metabolites are primarily excreted in urine [4].

Once administrated topically in eyes, almost 80% of the drops are drained through nasolacrimal duct to the nose, where they are absorbed systematically through nasal mucosa and it should be noted that as there is no hepatic first pass metabolism, the absorbed drug behaves like intravenous dose. Timolol can reach systemic circulation through conjunctival vessels as well. Timolol reaches peak concentration within 1 hour of topical administration with an average plasma concentration between 0.46 ng/mL and 1.38 ng/mL [4, 5]. A dose of one drop of 2.5–0.5% solution to each eye is equivalent to a 5–10 mg oral dose exposing the patient to adrenergic beta blocking effects and these include effects on CNS, pulmonary, cardiovascular, and endocrine systems [4–10].

Many reports are available in literature regarding Timolol Maleate's effects on heart rate and respiratory system. Some of them are particularly on interaction between Timolol and other cardiac drugs like beta blockers and calcium channel blockers [11, 12]. Others are those in which Timolol was the only cause found [13–17].

Although hypoglycemia or altered response to blood sugar control has been listed a few times in literature [17–19], topical beta blocker induced neurological side effects like confusion and psychosis are rare [20].

Our case report is unique in this regard where our patient developed three important side effects simultaneously and all of them improved after stopping the drug.

As pointed out earlier, cardiovascular effects have so far been extensively studied and reported [21, 22] and these include randomized control trials, crossover studies, or case reports but one thing should be noted that in most of these studies the participants included were healthy adults and they could not represent the real world patients which mostly consist of elderly patients with a number of comorbidities and most of them are already on either respiratory or cardiovascular medications. Moreover, these studies were limited to investigating the impact of Timolol Maleate in resting and peak heart rate during exercise but more serious side effects or outcomes like bradycardia or heart blocks were not assessed [23–26].

There is no specific antidote available for Timolol toxicity although there are some recommendations about the use of Glucagon 5–15 mg in slow intravenous infusion form if the patient does not respond to intravenous fluids and Atropine. In elderly patients, the effect of beta blockade has been found to be stronger and lasts longer [27].

Another important study was carried out by Pratt et al. [28] in which the researchers have looked at the association between ophthalmic Timolol and hospitalisation for bradycardia. This study summarizes some characteristics of patients admitted to hospital after initiation of Timolol. The major characteristics identified were mean age 82.6 and male sex preponderance 59.9%. It was also pointed out that the risk of symptomatic bradycardia was significantly increased in the 31st to 180 days after Timolol initiation.

## 3. Conclusion

Topically administrated beta blockers could have profound and prolonged systemic side effects especially in older age group of patients. Extra caution should be observed when prescribing glaucoma treatment to these patients and careful medical and drug history should be sought. If they are found to have other respiratory or cardiovascular comorbidities like ischemic heart disease or COPD, then glaucoma regimen should be carefully chosen. A close liaison between patient's physician and ophthalmologist is therefore necessary. A small dialogue among the treating physicians can save the patient from a potentially serious and life threatening situation. Acute care physicians including those in emergency and ICU should keep their angle of suspicion wide and if such patients are admitted with syncope or falls, systemic adverse drug reaction should be considered.
